# Supplementary material for: Optical Coherence Tomography Identifies Visual Pathway Involvement Earlier than Visual Function Tests in Children with MRI-Verified Optic Pathway Gliomas
Source: Cancers (Basel). 2022 Jan 9;14(2):318. doi: 10.3390/cancers14020318 (PMC8774215; doi:10.3390/cancers14020318)
Supplement: Supplementary file 1 [file cancers-14-00318-s001.zip › cancers-1476747-supplementary.pdf]

cancers

MDPI

Article

Optical Coherence Tomography Identifies Visual Pathway Involvement Earlier than Visual Function Tests in Children with MRI-Verified Optic Pathway Gliomas

Urszula Arnljots, Maria Nilsson, Ulrika Sandvik, Ida Hed Myrberg, Daniel Martin Munoz, Klas Blomgren and Kerstin Hellgren

Supplementary Material

Table S1. Clinical findings in 33 eyes with OPG that underwent all ophthalmological examinations and had pathological findings.

No Patient, Eye (R/L)	VA	Average GC-IPL thickness (µm)	Combined nasal sectors thickness (µm)	Combined temporal sectors thickness (µm)	VF affected	OPG location
1R	0.65	56	56.5	53	Nasal complete and temporal partial	3–4
1L	1.25	68	52	87	Temporal complete	4
2R	0.05	56	54	57.5	Temporal partial and superior nasal partial	3–5
2L	0.7	56	55.5	58	Temporal partial and nasal inferior partial	3–4
3R	0.6	73	65	82	Inferior temporal quadrant	3–4
3L	0.8	74	65	82	Normal	3–4
4R	1	66	59.5	72.5	Superior temporal partial	3–5
4L	1	64	60	65.5	Normal	3–5
5R	1	75	76.5	78	Normal	4
5L	1	75	72	80.5	Normal	4
6R	1	64	56.5	75	Inferior temporal complete and superior temporal partial	3–4
6L	1	62	60	63.5	Temporal partial and superior nasal partial	2–6
7R	0.4	56	53.5	61.5	Complete nasal and superior temporal partial	5–6
7L	0.7	60	61.5	54.5	Complete temporal and superior nasal partial	5–6
8R	0.2	49	48.5	47.5	Complete nasal and subtotal temporal	3–6
8L	1	53	42.5	65	Temporal complete	3–4
9R	0.3	72	66.5	77.5	Partial temporal and nasal	3–4
10R	0.7	58	58	55,5	Complete superior nasal, partial inferior nasal and partial superior temporal	1
11R	1	75	74.5	77	Normal	3–4
11L	1	75	74.5	77.5	Normal	4
12R	0.7	64	61	69	Enlarged blind spot	2–5
12L	0.8	64	57	73	Normal	2–5
13R	0.4	75	71.5	80.5	Partial temporal	4
13L	0.8	72	69.5	76.5	Partial temporal	1–5
14L	1	63	45	81	Complete temporal	4–5

15L	0.5	58	59.5	57	Diffuse central	1–2
16R	0.4	51	51	52.5	Central scotoma and complete temporal defect	3–4
17R	1	73	68.5	79.5	Normal	3–4
17L	1	73	68.5	79.5	Normal	3–4
18L	1	72	63	81.5	Normal	5
19R	1	67	64.5	73	Partial temporal	1–7
20R	0.9	57	54	62	Partial temporal	4
20L	0.16	54	55	52.5	Complete inferior temporal, partial superior temporal and inferior nasal temporal	1–4

Extension of tumors: (1) Intraconal (posterior boundary orbital apex); (2) Intracanalicular (posterior boundary optic foramen); (3) Intracranial-prechiasmatic; (4) Chiasmatic; (5) Optic tract; (6) Lateral geniculate nucleus; (7) Optic radiation. VA = visual acuity, L = left eye, R = right eye.
